# The Influence of Climate Change Efficacy Messages and Efficacy Beliefs on Intended Political Participation

**DOI:** 10.1371/journal.pone.0157658

**Published:** 2016-08-03

**Authors:** P. Sol Hart, Lauren Feldman

**Affiliations:** 1 Department of Communication Studies, University of Michigan, Ann Arbor, Michigan, United States of America; 2 School of Communication and Information, Rutgers University, New Brunswick, New Jersey, United States of America; Universidade de Vigo, SPAIN

## Abstract

Using an online survey experiment with a national sample, this study examined how changing the type and valence of efficacy information in news stories discussing global climate change may impact intended political participation through the mediators of perceived internal, external, and response efficacy. Overall, the results revealed that after a single exposure to a news story, stories including positive internal efficacy content increased perceived internal efficacy, while stories including negative external efficacy content lowered perceived external efficacy. There were limited impacts of other types of efficacy content on perceived efficacy. Perceived internal, external, and response efficacy all offered unique, positive associations with intentions to engage in climate change-related political participation. The results suggest that news stories including positive internal efficacy information in particular have the potential to increase public engagement around climate change. The implications for science communication are discussed.

## Introduction

Scientists widely agree that climate change is occurring, primarily caused by humans, and is already having significant negative impacts, which are expected to worsen over the next century and beyond [1–5]. Despite scholars’ recommendation that strong policy action is one of the most effective ways to address climate change [6], the U.S. government’s policy response has been relatively limited [7,8]. Scholars posit that citizen mobilization calling for new climate mitigation and adaptation policies is a critical ingredient for motivating government action [9]. However, political engagement on climate change by members of the public remains somewhat rare, with only about 10% of the U.S. public having contacted government officials to ask for action to be taken on the issue [10]. In light of this, communication scholars have paid increasing attention to how climate change is covered in the media and how different types of climate messages may influence public perceptions and engagement on the issue [8,11,12].

The present study examines a critical element that influences whether or not members of the public engage in political action around issues such as climate change: their sense of efficacy. Efficacy refers to individuals’ perception that a problem is addressable and that they are able to engage in the relevant action needed to address the problem [13]. Although perceived efficacy has been identified as an important variable in influencing political action in general [14] and environmental activism specifically [15], scant research has investigated how efficacy information as a variable in media messages may influence perceptions of efficacy and political action in the context of climate change [16]. In two media content analysis [11,17], we have developed a novel conceptualization of efficacy that combines elements from the Extended Parallel Process Model (EPPM) [18] and approaches taken in political science [13,14,19]. The resulting framework proposes three types of efficacy that are likely to be important determinants of public political engagement around climate change: internal, external, and response efficacy. The present study draws from this framework to examine how varying these three types of efficacy information in media stories influences corresponding perceptions of efficacy, and how these perceptions of efficacy, in turn, predict willingness to take political action on the issue of climate change. Overall, we find that all three types of efficacy beliefs have a positive influence on intended political action, but media messages do not necessarily influence all three types of efficacy perceptions.

### Literature Review

Previous research has suggested that climate change communication can be most effective by engaging in a dual role of encouraging public support for regulation to address climate change and fostering public engagement on the issue to build political pressure for the implementation of relevant policy [9]. While calls for changes in individual behavior, such as using less carbon intensive transportation or installing energy efficient lighting in households [20], carry merit as well, psychological barriers [21] and structural obstacles [22,23] make it difficult for individuals to voluntarily adopt such behaviors [9]. For example, an individual interested in using public transportation may find it difficult to do so, despite their intent, if the area they live in has poorly developed infrastructure. In contrast to calls for individual behavior change, government policies can help provide incentives for individuals to take action as well as address the structural issues that communities face to lower greenhouse gas emissions [9].

Multiple studies have examined how strategic messaging and news coverage may influence public support for climate mitigation policy and willingness to take political action on the issue [12,16,24–26]. While these studies have analyzed multiple message components, we are only aware of a single study that has examined how efficacy information may influence such engagement [16]. Efficacy, however, is a critical component in driving risk-related behavior change [27,28] and political participation [14,29–32], and the present study helps fill this research gap by examining how different types of efficacy information in news stories may influence perceptions of efficacy and, in turn, encourage or inhibit intended political action on climate change.

Some of the seminal work in understanding how efficacy is related to behavior was developed through Bandura’s self-efficacy theory [33]. This theory focuses primarily on two constructs: self-efficacy and outcome expectancy. Self-efficacy refers to the belief that one can engage in a behavior [33]. Outcome expectancy refers to the perceived likely outcome of engaging in a behavior. According to Bandura’s self-efficacy theory, when an individual perceives that they have the ability to engage in a behavior, and the individual believes that the behavior is likely to result in an outcome the individual desires, the individual is more likely to engage in the behavior [33]. This approach to understanding efficacy and behavior strongly influenced subsequent behavioral models, including the Extended Parallel Process Model (EPPM), which serves, in part, as the basis for the present study.

The role of efficacy information as a driver of behavior change is central to the EPPM [18], which offers predictions on the likely impacts of fear appeal messages. The EPPM posits that persuasive messages that convey a high level of threat–for example, regarding the risks of cigarette smoking or having unprotected sex–also must include efficacy information in order to induce attitude and behavior change conducive to averting the threat. Specifically, according to the EPPM, efficacy information helps individuals feel capable of overcoming a threat and, in turn, encourages protective action to lessen the threat; if efficacy is low, individuals will instead succumb to fear and engage in defensive mechanisms to control their emotions rather than take action to minimize the threat. In the EPPM, efficacy is comprised of two dimensions: self-efficacy, which refers to one’s ability to carry out the recommended behavior change, and response efficacy, which refers to the effectiveness of a particular action in averting a threat; these conceptualizations are similar, respectively, to the conceptualizations of self-efficacy and outcome expectancies in Bandura’s self-efficacy theory [33]. Other theories of behavior change also include parallel constructs. For example, in the Theory of Planned Behavior [34], behavioral beliefs are akin to response-efficacy, while perceived behavioral control is similar to self-efficacy.

The EPPM distinguishes between efficacy as a message characteristic and perceived efficacy. Several studies using the EPPM framework have shown that exposure to messages high in efficacy information heightens perceived efficacy [27,35–37]. These studies also have demonstrated that exposure to high efficacy messages results in attitudes and behavior consistent with the persuasive intent of the message [35,36] and that perceived efficacy is positively associated with message acceptance [27,37].

Despite the emphasis on efficacy in the EPPM, surprisingly few studies have simultaneously tested both the effects of efficacy message variables on perceived efficacy and the effects of perceived efficacy on behavioral intentions. Instead, the mediating role of perceived efficacy is assumed based on individuals’ attitudinal and behavioral responses to messages that are either high or low in efficacy in combination with a separate manipulation check that shows the effects of efficacy messages on perceived efficacy. Moreover, manipulations of efficacy in most EPPM studies tend to simultaneously vary both self and response efficacy [27,35]; thus, the unique influence of these two variables on efficacy perceptions and, in turn, behavior is relatively unknown. Thus, the present experiment–by independently manipulating different forms of efficacy information and examining their influence on perceptions and behavioral intentions–offers an important advance over previous research.

In the political domain, efficacy connotes the feeling that “political and social change is possible, and the individual citizen can play a part in bringing about this change” [38]. Political science scholars have differentiated between two different types of efficacy: internal efficacy, which refers to one’s personal sense that they can understand politics and act effectively in the political realm, and external efficacy, which reflects individuals’ beliefs about the government’s responsiveness to citizen demands [13,30,39]. These forms of efficacy have been found to be important motivators of general political participation [14,29–32], arguably because they help reduce the perceived costs and increase the perceived benefits of taking action in the political sphere. Myriad survey-based studies have linked exposure or attention to news and other forms of media to perceived internal and/or external political efficacy [40–44]. Still, based on these studies, it is difficult to know what exactly it is about media exposure that influences perceived efficacy. To our knowledge, ours is the first study to manipulate political efficacy information in climate change news stories in order to isolate its potential effects on perceived political efficacy and intended political behavior.

The present study utilizes our proposed conceptualization of efficacy for political issues such as climate change [11,17], which combines elements from the aforementioned approaches taken by the EPPM [18,45] and political science [13,19]. This approach examines three aspects of efficacy: internal efficacy, external efficacy, and response efficacy. As mentioned above, internal efficacy, which is very similar to the concept of self-efficacy in the EPPM, captures the ease with which individuals are able to take political action [30]. Indicators of internal efficacy include whether an individual feels they are knowledgeable enough about politics or a political issue and have the ability to take political action such as writing a letter to an elected official. However, even when an individual feels that they are able to take action such as writing a letter, a critical factor in whether they choose to do so is whether they believe that politicians themselves will actually respond to public engagement and input [46]. This second factor is referred to as external efficacy, which reflects individuals’ perception of whether elected officials will respond to public opinion and calls for policy change [14]. Finally, even if an individual believes that politicians will respond to calls for action, an additional factor is whether the political action that does occur will successfully address the issue. This final factor is referred to as response efficacy, which captures whether proposed policy action to address issues such as climate change will actually be effective in doing so [11,17,47].

In our initial study [17], we examined the representation of these three types of efficacy in US network TV news stories about climate change between 2005 and 2011. We also analyzed whether efficacy information was discussed in a positive way, suggesting that taking action would be possible and/or effective, or in a negative manner, noting obstacles to taking action or to the action’s success. Overall, the content analysis found that positive efficacy information was somewhat more prevalent than negative efficacy information; however, news stories were more likely to include both positive and negative efficacy information about climate change action than positive efficacy information alone. Further, coverage was heavily skewed towards talking about climate change action in terms of response efficacy, which was discussed in about a third of news stories. Both positive and negative information about internal efficacy and external efficacy was relatively absent. In a follow up study [11], which examined non-editorial news coverage in leading US newspapers, we found a similar pattern. While these content analyses offer insight into how efficacy information is represented in the news media, they do not inform how these types of media stories may influence public opinion on the issue, a research gap the present study helps fill.

The present study seeks to better understand how news stories about climate change may differentially influence public opinion and engagement when they focus on internal, external, and response efficacy. To the best of our knowledge, this question has only been examined in a previous study we conducted [16], which analyzed the influence of internal, external, and response efficacy information on emotional responses and intended political behavior in the context of climate change. Overall, this study found that positive efficacy information generally increased the feeling of hope while decreasing the feeling of fear. As both hope and fear were positively associated with political action on climate change, this led to countervailing indirect effects of efficacy information on intended climate activism.

The present study builds from our previous work [16], as well as previous research on the EPPM and in political communication, in several ways. First, the present study investigates the potential for climate change messages that emphasize different types of efficacy to shift public perceptions of efficacy, which past research [16] did not examine. In addition, our previous research in this area [16] only examined the influence of statements about factors that may facilitate efficacy; the present study expands on this approach by examining statements about factors that facilitate, as well as those that inhibit, efficacy around climate change (respectively referred to as positive and negative efficacy). To our knowledge, the present study also offers the first test of how perceptions of internal, external, and response efficacy may independently influence intentions to engage in political behavior related to climate change. Thus, the overall aim of the present study is to identify how positive or negative statements regarding internal, external, and response efficacy may influence intended political behavior as mediated by perceptions of these three types of efficacy.

Looking first to the influence of messages on perceptions of efficacy, we expect that positive and negative statements regarding the specific types of efficacy will influence perceptions of that type of efficacy in the respective direction. Thus, we formally propose the following hypotheses:

H1a: Exposure to positive internal efficacy content will increase perceptions of internal efficacy regarding climate change.
H1b: Exposure to negative internal efficacy content will lower perceptions of internal efficacy regarding climate change.
H2a: Exposure to positive external efficacy content will increase perceptions of external efficacy regarding climate change.
H3b: Exposure to negative external efficacy content will lower perceptions of external efficacy regarding climate change.
H3a: Exposure to positive response efficacy content will increase perceptions of response efficacy regarding climate change.
H3b: Exposure to negative response efficacy content will lower perceptions of response efficacy regarding climate change.

With regard to the potential influences of efficacy messages on non-corresponding types of efficacy perceptions (such as information about internal efficacy influencing perceptions of response efficacy), we ask as a formal research question:

RQ1: How will exposure to specific types of efficacy content influence perceptions of other types of efficacy?

As perceptions of efficacy have been positively linked with willingness to take political action in general, we likewise expect that greater perceptions of internal, external, and response efficacy will be positively linked with greater intentions to take political action around climate change. Thus, we formally state:

H4a: Perceptions of internal efficacy will be positively associated with intentions to take political action on climate change.
H4b: Perceptions of external efficacy will be positively associated with intentions to take political action on climate change.
H4c: Perceptions of response efficacy will be positively associated with intentions to take political action on climate change.

Finally, we predict a significant mediation effect, such that exposure to information about efficacy will significantly impact intentions to take political action indirectly through changes in efficacy perceptions. Formally stated:

H5: Exposure to efficacy content in news stories will influence intentions to take political action through the mediators of internal, external, and response efficacy perceptions.

**Fig 1:**
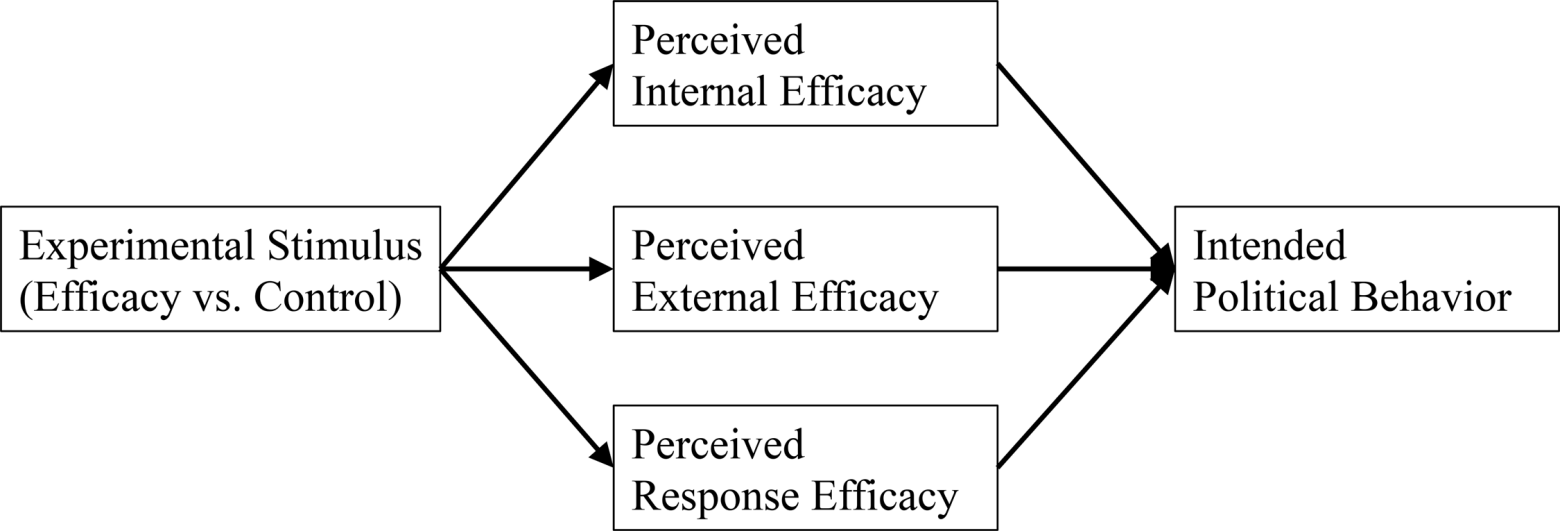
Conceptual Map for Testing.

See Fig 1 for a diagram of our hypothesized model.

## Method

The study hypotheses and research question were tested using an experiment embedded within an online survey. This study was approved by the Institutional Review Board (IRB) at the University of Michigan (HUM00082574). All participants consented to participate through an online consent form, and the University of Michigan IRB approved this procedure. A sample of 1,426 participants was recruited through Qualtrics panels. Qualtrics draws survey respondents from traditional, actively managed market panels. Participants are recruited through e-mail sign-up, web banners, social media, and invitation only methods. Quota targets for age, gender, and race and ethnicity were utilized to ensure that the participant pool used in this experiment approximated census estimates. For age, participant quota targets were set for even quartiles across the following four age groups: 18–34, 35–44, 45–54, 55+; for gender, quota targets were set at a 50/50 gender split; for race and ethnicity, quota targets were set to 67% White, 13% African American, 10% Hispanic, and 10% other.

Participants were randomly assigned to one of seven experimental conditions: either a no-message control condition or one of six treatment conditions. The number of participants in each condition ranged from 200 to 208 individuals. All of the treatment conditions consisted of an exposure to a constructed news story that was indicated to be from the Associated Press. The news story discussed the likely impacts of climate change, including heat waves, floods, droughts, extreme weather, and effects on human health in the United States. The treatment conditions varied according to the type of efficacy information emphasized in the news story (internal, external, or response) and the valence of this information (positive vs. negative). The six treatment conditions were 1) positive internal efficacy, 2) negative internal efficacy, 3) positive external efficacy, 4) negative external efficacy, 5) positive response efficacy, and 6) negative response efficacy. In order to manipulate efficacy information, we varied the headline of the news articles and the second paragraph of the three-paragraph news article, which described policy actions that may be taken to respond to climate change. Based on word count, the efficacy manipulation comprised about 40% of the article text. To improve generalizability, we used stimulus sampling to vary the type of policy action proposed in the treatment articles: For the roughly 200 participants who were assigned to a given efficacy treatment condition, a random half saw a description of a carbon tax as a policy response and a random half saw a description of investment in renewable energy as a policy response.

For the efficacy treatment manipulation, a positively valenced efficacy condition included text suggesting that efforts to address climate change would be successful in the respective efficacy domain. For the internal, external, and response efficacy types, respectively, this included text suggesting that many individuals are able to take political action on climate change, that the U.S. government is responsive to public calls for action on climate change, or that U.S. government policies can help stop negative effects of climate change from occurring. The negative efficacy conditions called into question the feasibility or effectiveness of action. For the internal, external, and response efficacy types, respectively, this text indicated that many Americans find it difficult to take political action on the issue, that the U.S. government is not responding to public calls for action, or that U.S. government policies are unlikely to stop the negative impacts of climate change from occurring. An example of one of the stimulus stories is included in S2 File.

### Variables

#### Experimental conditions

As mentioned in the methods section, participants were randomly exposed to one of seven conditions: 1) a no-message control condition, 2) a positive internal efficacy condition, 3) a negative internal efficacy condition, 4) a positive external efficacy condition, 5) a negative external efficacy condition, 6) a positive response efficacy condition, or 7) a negative response efficacy condition.

#### Mediators

Three mediating variables were measured: perceived internal efficacy, perceived external efficacy and perceived response efficacy for political action to address climate change.

Perceived internal efficacy was measured by adapting established measures of internal political efficacy [30,39] to the specific context of climate change. Previous research found that items assessing citizens’ perceived understanding of political issues, their sense of being well-informed about politics, and their feeling of being well-qualified to participate in politics together perform as a valid and reliable measure of internal efficacy [30,39]. We directly adapted these items to the issue of climate change and also included additional items to help further capture one’s perceived ability to understand and participate in politics in the context of climate change. Given that climate change is both a scientific and political issue, internal political efficacy in the realm of climate change requires confidence in one’s understanding of politics in general, one’s understanding of politics as it applies to climate change specifically, and one’s understanding of climate change itself, as well as in one’s ability to effectively participate on the issue of climate change. Thus, we asked participants how much they agreed or disagreed with the following statements: 1) “I think that I am as well informed about politics and government as most people,” 2) “I think that I know as much about the politics surrounding climate change as most people,” 3) “I think that I am as well informed about government policies that could address climate change as most people,”4) “I consider myself to be well qualified to participate in politics related to climate change,” 5) “I feel that I have a pretty good understanding of climate change,” and 6) “I feel confident that, if I wanted to, I would have the ability to contact a government official about climate change.” Each item was rated on a 7-point scale that ranged from strongly disagree (1) to strongly agree (7). The respective answers were then averaged into a single internal efficacy scale (Cronbach’s alpha = .92, M = 4.48, SD = 1.38).

Perceived external efficacy was measured by asking participants how much they agreed or disagreed with the following statements: 1) “People like me don’t have any say about what the government does about climate change,” 2) “Public officials don’t care much about what people like me think about climate change,” and 3) “The government pays attention to what people like me think when they decide what to do about climate change.” These items were adapted from established measures of external political efficacy [30,39] to the specific context of climate change. Each item was rated on a 7-point scale that ranged from strongly disagree (1) to strongly agree (7). Items (1) and (2) were reverse coded, and the three items were then averaged into a single external efficacy scale (Cronbach’s alpha = .75, M = 3.28, SD = 1.35).

Perceived response efficacy was measured by asking participants how much they agreed or disagreed with the following statements: 1) “If government officials were to pass laws to reduce America’s dependence on fossil fuels, this would be effective in reducing the negative impacts of climate change,” and 2) “If government officials were to pass laws to reduce America’s dependence on fossil fuels, this would be effective in reducing greenhouse gas emissions.” Each item was rated on a 7-point scale that ranged from strongly disagree (1) to strongly agree (7). The two items were then averaged into a single response efficacy scale (r = .86, M = 4.75, SD = 1.58).

To confirm that the items used to measure internal efficacy, external efficacy, and response efficacy loaded on three distinct factors, we conducted a principal components factor analysis using varimax rotation of all 11 efficacy items. The analysis revealed three distinct factors and confirmed that the indicators for each of the three types of efficacy loaded cleanly onto their respective factors. The first factor (internal efficacy) had an eigenvalue of 4.34 and explained 39.5% of the variance; the second factor (external efficacy) had an eigenvalue of 2.31 and explained 21.0% of the variance, and the third factor (response efficacy) had an eigenvalue of 1.56 and explained 14.2% of the variance. All items had a final primary factor loading over .7 and did not load on another factor at above .2.

#### Dependent variable

Intended climate change-related political participation was measured by asking participants how likely or unlikely it is that in the next twelve months they will engage in the following activities: 1) “contact government officials to urge them to take action to reduce climate change,” 2) “participate in a rally or protest in support of action to reduce climate change,” 3) “sign a petition in support of taking action to reduce climate change,” 4) “join or volunteer with an organization working to reduce climate change,” and 5) “donate money to an organization working to reduce climate change.” The likelihood of each activity was answered separately on a 7-point scale that ranged from very unlikely (1) to very likely (7). The respective answers were then averaged together to create a single political behavior scale (Cronbach’s alpha = .92, M = 3.45, SD = 1.64).

#### Control variables

The regression analysis controlled for the sociodemographic variables of age, gender, education, and income, as well as political ideology and ecological beliefs. The analysis also controlled for the policy type stimulus sample (i.e., carbon tax or renewable energy). Age was measured by asking participants to enter their age on their last birthday (M = 45.58, SD = 15.24). Gender was measured by asking participants whether they were Male (1) or Female (2) (52% female). Education was measured by asking participants to indicate they highest level of education on a 10-point scale that ranged from Grade 8 or lower (1) to Doctorate degree (PhD, etc.) (10) (Median = 5, “Some college, no degree,” M = 5.45, SD = 1.72). Income was measured by asking participants what their annual household income was on a 9 point scale that ranged from less than $20,000 (1) to $160,000 or higher (9) (Median = 3, $40,000–$59,999, M = 3.15, SD = 1.94).

Political ideology was measured by asking participants to indicate, generally speaking, how they would best describe their political views on a 7-point scale that ranged from very liberal (1) to very conservative (7) with “moderate or middle of the road” used as the midpoint (4) (M = 3.96, SD = 1.57).

Ecological beliefs were measured using a well established scale [48], which asks participants how much they agree or disagree with the following statements: 1) “The balance of nature is very delicate and easily upset by human activities,” 2) “Ecological, rather than economic, factors must guide our use of natural resources,” 3) “We attach too much importance to economic measures of well being in our society,” 4) “We are approaching the limit of the number of people the earth can support,” 5) “When humans interfere with nature, it often produces disastrous consequences,” 6) “Humans must live in harmony with nature in order to survive,” 7) “There are limits to growth beyond which our industrialized society cannot expand.” Each question was answered on a 7-point scale that ranged from strongly disagree (1) to strongly agree (7). The items were averaged into a single ecological beliefs scale (Cronbach’s alpha = .85, M = 5.11, SD = 1.09).

### Analysis

The analysis was conducted in two phases. First, a one-way ANOVA with 1-sided Dunnett post-hoc tests comparing the treatment conditions to control was conducted to investigate how differences in message content affected the three respective mediators of perceived internal, external, and response efficacy. Then the mediation model was analyzed with SPSS version 21 and the SPSS PROCESS macro version 2.15 [49]. The PROCESS macro can be downloaded for free from http://processmacro.org/download.html. The mediation analysis tested whether the message treatment conditions, as compared to the control group, influenced the three mediators of internal, external, and response efficacy, and how these types of efficacy, in turn, influenced political behavior. The PROCESS macro utilizes a regression based path analytic framework to test hypothesized direct and indirect effects. In addition to OLS regression coefficients, the PROCESS macro offers bootstrapped confidence intervals for the indirect effects of the experimental conditions on political behavior through perceptions of internal, external, and response efficacy, while accounting for all control variables. The use of bootstrapped confidence intervals is generally considered superior to alternatives such as the Sobel test or causal steps approach [49]. Bootstrapped intervals were calculated using 10,000 sampling iterations and bias-corrected estimates. The 95% confidence intervals may be interpreted such that if the lower and upper confidence intervals are either both above 0 or both below 0 there is a statistically significant effect, whereas if they straddle 0 there is not a significant effect.

## Results

We first examined how the experimental conditions influenced the mediators of internal, external, and response efficacy with a one-way ANOVA using 1-sided Dunnett post-hoc tests comparing each condition to control. The means of perceived internal, external, and response efficacy by condition are provided in Table 1. Looking first to the influence on perceptions of internal efficacy, the overall test of between subject effects was significant (F (6, 1,419) = 3.75, p < .001). Post-hoc investigations revealed that the positive internal efficacy condition increased perceptions of internal efficacy compared to the control condition (p < .05), in support of H1a. No other condition significantly shifted perceptions of internal efficacy compared to control. Thus, the results failed to support H1b, which predicted that negative internal efficacy information would lower perceived internal efficacy.

**Table 1:** Mean Perceived Internal, External, and Response Efficacy by Experimental Condition.

Experimental Condition	Perceived Internal Efficacy	Perceived External Efficacy	Perceived Response Efficacy
Control	4.51 (1.45)	3.35 (1.30)	4.74 (1.59)
Positive internal efficacy	**4.87 (1.34)**	3.58 (1.42)	5.10 (1.58)
Positive external efficacy	4.49 (1.30)	3.36 (1.44)	4.72 (1.65)
Positive response efficacy	4.34 (1.29)	3.16 (1.30)	4.83 (1.57)
Negative internal efficacy	4.37 (1.45)	3.18 (1.36)	4.55 (1.63)
Negative external efficacy	4.35 (1.36)	**3.02 (1.19)**	4.74 (1.48)
Negative response efficacy	4.42 (1.40)	3.28 (1.33)	4.54 (1.51)

*Note*: Boldfaced text is used to denote significant effects compared to control, p < .05. Standard deviations are provided in parentheses.

Looking next to external efficacy, the overall test of between subject effects was significant (F (6, 1419) = 3.64, p < .001). The 1-sided Dunnett post-hoc analysis showed that the negative external efficacy condition led to significantly lower perceptions of external efficacy compared to control (p < .05), in support of H2b. As no other condition significantly shifted perceived external efficacy compared to control, the results failed to support H2a, which predicted that positive external efficacy information would increase perceptions of external efficacy.

Looking to response efficacy, the overall test of between-subject effects indicated a significant difference between conditions in influencing perceptions of response efficacy (F = (6, 1419) = 2.93, p < .01). A1-sided Dunnett post-hoc test did not reveal any significant differences between the control condition and the individual treatment conditions, failing to support H3a and H3b, both of which predicted an influence of response efficacy information on perceptions of response efficacy.

PROCESS was utilized to investigate whether the respective experimental conditions, as compared to the control condition, had any mediated effects on intended political participation through the three types of perceived efficacy. Looking first to the influence of the experimental conditions on perceived efficacy from the PROCESS regression analysis (see Table 2), the positive internal efficacy condition significantly increased perceived internal efficacy (unstandardized b = .31, p < .05), the negative external efficacy condition significantly reduced perceived external efficacy (b = -.30, p < .05), and the positive internal efficacy condition significantly increased perceived response efficacy (b = .29, p < .05); no other relationships between the treatment conditions and perceived efficacy were significant. Looking next to the influence of the three types of efficacy on intentions to engage in political behavior (see Table 2), perceived internal (b = .28, p < .001), external (b = .29, p < .001), and response (b = .37, p < .001) efficacy all had significant positive associations with political behavior, in support of H4a, H4b, and H4c. Finally, looking to the mediated influence of the treatment conditions, through perceived efficacy, onto intended political behavior (see Table 3), the positive internal efficacy condition had significant positive indirect effects through perceived internal efficacy (b = .09, lower level confidence interval (LLCI) = .016, upper level confidence interval (ULCI) = .17) and response efficacy (b = .11, LLCI = .023, ULCI = .20), while the negative external efficacy condition had a significant negative indirect effect through perceived external efficacy (b = -.08, LLCI = -.17, ULCI = -.013). No other mediated relationships were significant, offering partial support for H5.

**Table 2:** Ordinary Least Squares Regression Results Predicting Efficacy Perceptions and Intended Political Behavior.

Predictor Variables	Internal Efficacy	External Efficacy	Response Efficacy	Political Behavior
Constant	.363 (.31)***	3.73 (.32)***	2.97 (.30)***	-.1.60 (.33)***
*Experimental Treatment Conditions*				
Positive internal	.31 (.13)*	.23 (.13)	.29 (.12)*	.02 (.12)
Positive external	-.05 (.13)	.02 (.13)	.05 (.12)	.06 (.12)
Positive response	-.14 (.13)	-.17 (.13)	.18 (.12)	.07 (.12)
Negative internal	-.16 (.13)	-.16 (.13)	-.06 (.12)	.26 (.12)*
Negative external	-.14 (.13)	-.30 (.13)*	.10 (.12)	.20 (.12)
Negative response	-.08 (.13)	-.06 (.13)	-.15 (.12)	.23 (.12)
*Perceived Efficacy*				
Internal efficacy	-	-	-	.28 (.03)***
External efficacy	-	-	-	.29 (.03)***
Response efficacy	-	-	-	.37 (.03)***
*Control Variables*				
Age	.01 (.002)***	-.005 (.002)*	-.02 (.002)***	-.003 (.002)
Gender (Male)	-.54 (.07)***	.12 (.07)	-.04 (.07)	.11 (.07)
Education	.14 (.02)***	.02 (.02)	.001 (.02)	-.05 (.02)*
Income	.07 (.02)***	.04 (.02)	.03 (.02)	-.002 (.02)
Political ideology	-.01 (.02)	-.08 (.03)**	-.24 (.02)***	-.09 (.02)***
Environmental values	.05 (.03)	-.05 (.04)	.67 (.03)***	.29 (.04)***
Action type (Carbon Tax)	-.007 (.07)	-.11 (.07)	-.22 (.07)***	.11 (.07)
R^2^	0.14	0.04	0.41	0.44
*N*	1419	1419	1419	1419

*Note*: Unstandardized regression coefficients are reported. Investment in Renewable Energy is the reference category for each action type. Standard errors are provided in parentheses.

***p < .001

**p < .01

*p < .05.

**Table 3:** Indirect Effects of the Efficacy Message Type on Intended Political Behavior via Perceived Efficacy.

	Mediators					
	Internal Efficacy		External Efficacy		Response Efficacy	
Condition	Indirect Effect (*Boot SE*)	Boot 95% CI	Indirect Effect (*Boot SE*)	Boot 95% CI	Indirect Effect (*Boot SE*)	Boot 95% CI
Positive internal efficacy	**.09 (.04)**	**[.02, .17]**	.07 (.04)	[-.004, .15]	**.11 (.04)**	**[.02, .20]**
Positive external efficacy	-.01 (.04)	[-.08, .06]	.006 (.04)	[-.07, .08]	.02 (.04)	[-.06, .11]
Positive response efficacy	-.04 (.04)	[-.11, .04]	-.05 (.04)	[-.12, .03]	.07 (.04)	[-.02, .16]
Negative internal efficacy	-.04 (.04)	[-.11, .04]	-.05 (.04)	[-.12, .04]	-.02 (.05)	[-.11, .08]
Negative external efficacy	-.04 (.04)	[-.11, .04]	**-.09 (.04)**	**[-.16, -.01]**	.04 (.04)	[-.05, .12]
Negative response efficacy	-.02 (.04)	[-.10, .05]	-.02 (.04)	[-.09, .06]	-.05 (.05)	[-.15, .04]

*Note*: Bootstrapped standard errors and confidence intervals (CIs) were computed using 10,000 bootstrap samples. Boldface text is used to denote significant effects, p < .05. All treatment conditions are compared to the control condition.

## Discussion

This experiment adds to the growing literature on how climate change messages are associated with perceptions and engagement on the issue [8,26,50,51]. Overall, the results of the experiment offered mixed support for the proposed hypotheses. Positive internal efficacy information increased perceptions of internal efficacy, in support of H1a, and negative external efficacy information lowered perceptions of external efficacy, in support of H2b. The other predicted influences of mediated efficacy content on efficacy perceptions, however, were not observed, failing to support H1b, H2a, H3a, and H3b. We also note that the OLS regression from the mediation analysis suggested a positive influence of internal efficacy information on perceptions of response efficacy, but this result should be treated cautiously as the Dunnett post-hoc examination of this relationship, which corrects for family-wise error, did not reveal a significant relationship. Thus, in the investigation of RQ1, which asked whether specific types of efficacy information in media stories may influence other types of efficacy perceptions, we did not find strong evidence of this effect occurring (although the relationship between internal efficacy information and response efficacy perceptions may be worth exploring in future examinations).

The present study makes an important contribution to our understanding of the relationship between efficacy and intended political participation by demonstrating that all three types of perceived efficacy examined here–internal, external, and response–have unique, significant positive associations with intentions to become politically engaged on the issue of global climate change. This study is the first we are aware of to empirically test the conjoint influence of these three efficacy constructs on intended political participation in the context of climate change, and the significant observed effects are a strong indicator that it would be beneficial to employ this conceptualization of efficacy in future studies. In support of this work, the present study offers a set of survey items that load well on the three efficacy factors and that may be used directly in future studies regarding climate change as well as adapted to other sociopolitical issues.

Finally, looking at overall mediation effects, the mediation analysis found that positive response efficacy information significantly increased intentions to engage in political action through the mediators of perceived internal and response efficacy, while negative external efficacy information significantly reduced intentions to engage in political action through the mediator of perceived external efficacy. No other mediation effects were significant.

The results of the present study demonstrate the difficulty that may exist in shifting perceived efficacy on the issue of climate change. Climate change is increasingly polarized along political lines [52], which can lead to hardened opinions that are resistant to change [8]. Also, given that some scholars have characterized political efficacy as a relatively stable psychological trait [32], perceptions of efficacy may not be vulnerable to short-term change as the result of a single message exposure, as was utilized in this experiment. In particular, our manipulation may not have been strong enough to change perceptions of efficacy cultivated by long-term exposure to media coverage about climate change, which tends not to emphasize efficacy information [11,17]. We note that the stimuli materials were entirely text based, and did not utilize images, which some previous work has suggested may shift efficacy perceptions [53]. It is also possible that messages that use rich narratives to facilitate involvement with characters and narrative transportation [54] may be more effective in shifting efficacy perceptions as well. Finally, a manipulation incorporating repeated exposure, rather than a single exposure, to efficacy information may show different results. Future work may benefit by utilizing various text, imagery, and narrative combinations, in additional to different levels of exposure, to assess how these may affect efficacy perceptions and engagement.

As mentioned above, messages that included positive internal efficacy information indirectly increased intentions to take political action; however, overall there was a limited effect of the media messages on perceived efficacy. Based on the Dunnett post-hoc tests, only two of the six experimental conditions, as compared to control, significantly shifted efficacy perceptions. One of these findings, the influence of negative external efficacy on perceived external efficacy, should be treated with some caution as well, as the R^2^ from the regression predicting perceived external efficacy was very low, at .04, indicating that the predictive variables had limited explanatory power. Nevertheless, the present study does find that when efficacy perceptions do increase they are significant predictors of intended political behavior; internal, external, and response efficacy all had strong positive associations with issue relevant political behavior while controlling for the other types of efficacy. Thus, future work should continue to develop and test media messages and other strategies that may more effectively increase perceptions of political efficacy in the context of climate change.

What implications do these results have for climate change communication? This study suggests that messages including positive internal efficacy information have the potential to indirectly increase political engagement on the issue. This is important information for strategic communicators working in the area of climate change. For example, the results of this study strongly suggest that communication efforts may benefit not only by focusing on the possible effects of different policy responses to climate change, but also by describing specific steps that interested members of the public may take in the political sphere. The results also can help journalists better understand the implications of how they choose to cover climate change for public opinion and behavior. We note again that in our previous work [11,17], we found that when journalists report on the efficacy of actions to address climate change, the stories are heavily weighted towards discussing whether or not these actions will be successful in mitigating climate change (response efficacy) and include very limited information on the ability of individuals to take specific actions (internal efficacy). The present study suggests that media coverage that weights the three types of efficacy more evenly, and specifically includes positive information about internal efficacy, may be more likely to promote public engagement on the issue. While we recognize that many journalists see the purpose of the news media as primarily to inform, rather than persuade, there are multiple ways that a journalist may cover a story in a normatively acceptable manner, and it is important to understand how these choices may “nudge” [55–57] individuals to become more or less engaged on important sociopolitical issues such as global climate change. Thus, the findings of the present study offer journalists information on how the distribution of different types of efficacy in their reporting may influence and engage their audiences.

Strengths of this study include a carefully controlled experimental design and a diverse participant sample. As with any study, there are limitations that should be taken into account. This is only a single study, and caution should be taken when generalizing. In particular, it is unclear how the results would generalize outside the relatively artificial conditions of the present experiment; the effects of efficacy messages may be altered under the noisier conditions of real-world communication environments. In addition, the dependent variable utilized a self-report of political engagement intentions, but did not measure actual behavior; thus, the present study cannot offer direct implications on how exposure to efficacy information may influence actual behavior.

Overall, the data presented here suggest that the conceptualization of efficacy into three types–internal, external, and response–is a useful approach for understanding how efficacy may be associated with political engagement on sociopolitical issues such as climate change. In addition, while media messages containing efficacy information, in general, may have limited effects on perceptions of efficacy regarding climate change, there is evidence that messages that include positive internal efficacy information, in particular, have the potential to increase engagement on the issue by increasing efficacy perceptions.

## Supporting Information

S1 File Dataset. (SAV)

S2 File Sample Media Story–Positive Internal Efficacy Condition. (TIFF)
