# Supplementary figures and images for: The Influence of Climate Change Efficacy Messages and Efficacy Beliefs on Intended Political Participation
Source: PLoS One. 2016 Aug 3;11(8):e0157658. doi: 10.1371/journal.pone.0157658 (PMC4972420; doi:10.1371/journal.pone.0157658)

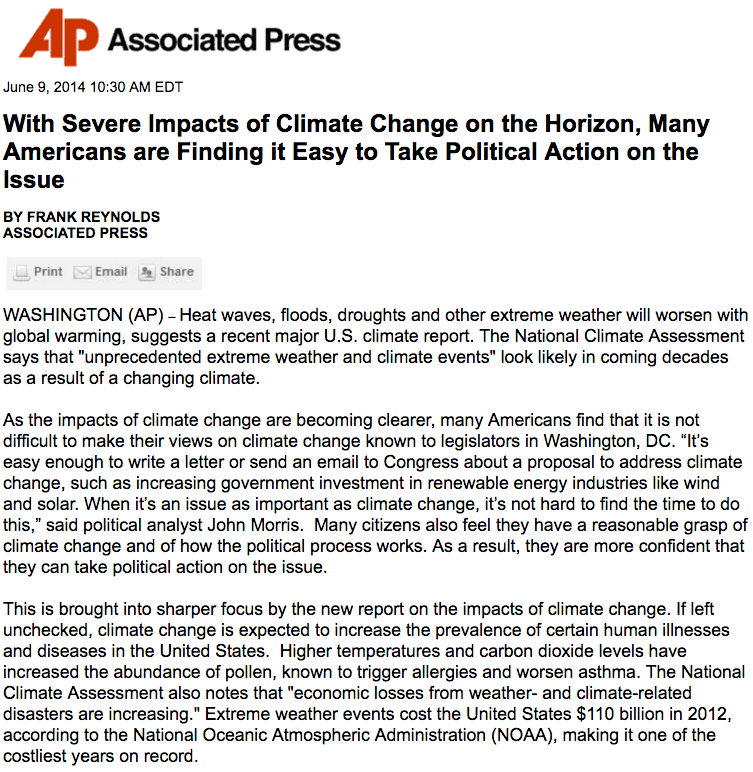

Supplement: S2 File — (TIFF) [file pone.0157658.s002.tiff]
